# Diffusion Tensor Imaging as Neurologic Predictor in Patients Affected by Traumatic Brain Injury: Scoping Review

**DOI:** 10.3390/brainsci15010070

**Published:** 2025-01-14

**Authors:** Federica Paolini, Salvatore Marrone, Gianluca Scalia, Rosa Maria Gerardi, Lapo Bonosi, Umberto Emanuele Benigno, Sofia Musso, Alba Scerrati, Domenico Gerardo Iacopino, Francesco Signorelli, Rosario Maugeri, Massimiliano Visocchi

**Affiliations:** 1 Neurosurgical Clinic, AOUP “Paolo Giaccone”, Post Graduate Residency Program in Neurologic Surgery, Department of Biomedicine Neurosciences and Advanced Diagnostics, School of Medicine, University of Palermo, 90127 Palermo, Italy; rosamariagerardimd@gmail.com (R.M.G.); lapo.bonosi@gmail.com (L.B.); umbertobenigno@gmail.com (U.E.B.); sofiamusso.sm@gmail.com (S.M.); gerardo.iacopino@unipa.it (D.G.I.); rosario.maugeri1977@gmail.com (R.M.); 2 Unit of Neurosurgery, Sant’Elia Hospital, 93100 Caltanissetta, Italy; salvo.mr89@gmail.com; 3 Neurosurgery Unit, Department of Head and Neck Surgery, ARNAS Garibaldi, 95124 Catania, Italy; gianluca.scalia@outlook.it; 4 Department of Translational Medicine, University of Ferrara, 44121 Ferrara, Italy; alba.scerrati@unife.it; 5 Department of Neurosurgery, Sant’Anna University Hospital of Ferrara, 44124 Ferrara, Italy; 6 Fondazione Policlinico Universitario Agostino Gemelli IRCCS, Università Cattolica del Sacro Cuore, 00168 Rome, Italy; francesco.signorelli@policlinicogemelli.it; 7 CVJ Operative Unit, CVJ Research Center Università Cattolica del Sacro Cuore, Fondazione Policlinico Universitario Agostino Gemelli IRCCS, 00168 Rome, Italy; mvisocchi@hotmail.com

**Keywords:** traumatic brain injury (TBI), diffuse axonal injury (DAI), diffusion tensor imaging (DTI), white matter (WM)

## Abstract

**Background**: Diffusion tensor imaging (DTI), a variant of Diffusion Weighted Imaging (DWI), enables a neuroanatomical microscopic-like examination of the brain, which can detect brain damage using physical parameters. DTI’s application to traumatic brain injury (TBI) has the potential to reveal radiological features that can assist in predicting the clinical outcomes of these patients. What is the ongoing role of DTI in detecting brain alterations and predicting neurological outcomes in patients with moderate to severe traumatic brain injury and/or diffuse axonal injury? **Methods**: A scoping review of the PubMed, Scopus, EMBASE, and Cochrane databases was conducted according to the Preferred Reporting Items for Systematic Reviews and Meta-Analysis (PRISMA) guidelines. The aim was to identify all potentially relevant studies concerning the role of DTI in TBI. From an initial pool of 3527 publications, 26 articles were selected based on relevance. These studies included a total of 729 patients with moderate to severe TBI and/or diffuse axonal injury. DTI parameters were analyzed to determine their relationship with neurological outcomes post-TBI, with assessments of several brain functions and regions. **Results**: The studies included various DTI parameters, identifying significant relationships between DTI variations and neurological outcomes following TBI. Multiple brain functions and regions were evaluated, demonstrating the capability of DTI to detect brain alterations with higher accuracy, sensitivity, and specificity than MRI alone. **Conclusions**: DTI is a valuable tool for detecting brain alterations in TBI patients, offering enhanced accuracy, sensitivity, and specificity compared to MRI alone. Recent studies confirm its effectiveness in identifying neurological impairments and predicting outcomes in patients following brain trauma, underscoring its utility in clinical settings for managing TBI.

## 1. Introduction

Traumatic Brain Injury (TBI) is one of the most common neurological disorders. TBI also involves huge social weight. Even if the most relevant clinical alterations in TBI patients develop in the acute phase, the chronic phase is not to be underestimated, as it encompasses a range of neurological sequelae with long-term consequences, such as an increased risk of neurodegeneration [1,2].

Diffusion tensor imaging (DTI), a variant of Diffusion Weighted Imaging (DWI) provides a neuroanatomical microscopic-like study capable of detecting brain damages by using physical parameters that can detect the structural integrity of axons and the water diffusivity inside [1].

In current practice, DTI is a technique that boosts MRI in terms of detecting in vivo microstructural alterations of white matter tracts. Its application in patients who have experienced a TBI is helpful in disclosing imaging features of pathophysiological mechanisms such as myelin degeneration, Wallerian degeneration, contraction, and collapse in axonal structure. Being highly sensitive to axonal integrity alterations, DTI metrics alterations may appraise changes in white matter (WM) density and the directionality of water along nerve fibers, showing axonal lesions undetectable by means of CT or conventional MRI scans. Literature reports have also highlighted the association between changes in DTI values with objective measures of complex cerebral functions, which may help in forecasting patient outcome [2].

Despite DTI not being routinely performed in TBI patients, a deeper understanding of its potentialities in terms of outcome prediction could be useful to assess which patients could potentially benefit from a prompt MRI evaluation as a means of addressing specific therapeutic efforts, as this already happens in stroke management. A comprehensive disclosure of future perspectives implies the knowledge of the state of the art, thus the revision of the literature is meant to explore the ongoing application of DTI regarding usefulness and prognostic potentialities, among which is the tailoring of patient care after brain trauma.

## 2. Materials and Methods

This study was conducted in accordance with the PRISMA guidelines [3]. An extensive scoping search was conducted on the PubMed, Scopus, EMBASE, and Cochrane databases to identify all potentially relevant studies regarding the role and usefulness of diffusion tensor imaging (DTI) in traumatic brain injury (TBI).

Many quantitative parameters and scalar matrices can be extracted and evaluated using the DTI. The keywords and their significance are reported from the literature below:

1. Fractional anisotropy (FA) is a scalar value between zero and one that describes the degree of anisotropy of a diffusion process. FA assumes values in a range from 0 (completely isotropic diffusion) to 1 (fully anisotropic, unidirectional diffusion). In this context, some authors have proposed using FA as a potential biomarker of axonal integrity [4].
2. Mean diffusivity (MD) quantifies cellular and membrane density. An increase in MD indicates pathological processes such as edema or necrosis, according to [5].
3. The apparent diffusion coefficient (ADC) value is a quantitative measure of the degree of impedance encountered by the diffusion of water molecules, enabling a more accurate definition of tissue reodynamic and pathological conditions, such as stroke, brain edema, or tumors.
4. Radial diffusivity (RD) represents the apparent diffusion coefficient in the direction perpendicular to axonal fibers, and its increase could be used as biomarker for microstructural damage or demyelination processes [6].
5. Axial diffusivity (AD) refers to the magnitude of diffusion parallel to fiber tracts, and its decrease might reflect axonal injury, reduced axonal caliber, or a less coherent orientation of axons. Some authors believe that AD assumes different regional values depending on the severity of the trauma [7].

The reference sections of included articles were also screened for additional relevant studies. A search of the literature was conducted from March to April 2024 using the following search strings:

- DTI AND traumatic brain injury: 749 articles;
- DTI AND brain trauma: 735 articles;
- DTI AND brain trauma AND outcome: 135 articles;
- DTI AND traumatic brain injury AND outcome: 143 articles;
- DTI AND brain recovery: 346 articles;
- Tracts AND brain trauma: 635 articles;
- DTI AND diffuse axonal injury: 78 articles;
- MR AND traumatic brain injury: 623 articles;
- DAI (diffuse axonal injury) AND MR intensity variations: 1 article.

Three authors (L.B., U.E.B., and S.M.) independently conducted the abstract screening for eligibility. Any discordance was resolved by consensus with a third senior author (F.P.)

The first stage of selection focused on identifying studies assessing the usefulness of DTI sequences in traumatic brain injury patients and their role in predicting neurological function after trauma. Neurological outcomes were evaluated using the Glasgow Coma Scale (GCS) and assessments of complex cerebral functions. Only moderate to severe traumatic brain injury patients were considered, including those with complications such as DAI, acute subdural/extradural hematomas, and others. The need for neurosurgical intervention was not an inclusion criterion, as both surgical and non-surgical patients were included. Our search included articles from 2015. This year corresponds to the period in which the scientific literature started to focus more on the topic, as shown by the increasing number of publications related to the topic.

Papers were selected according to the following inclusion criteria:

- Full-text articles available;
- English language only;
- Patients older than 18 years with a history of head trauma;
- Use of DTI sequences to detect brain alterations in moderate to severe traumatic brain injury;
- Evaluation of neurological outcomes (no restrictions on the timing of the evaluation after trauma);
- Articles published from 2015 onwards.

The exclusion criteria included the following:

- Full-text articles in languages other than English;
- Studies focused on patients with mild traumatic brain injury;
- Patients younger than 18 years;
- No available data on neurological outcomes;
- Studies not utilizing DTI MRI sequences.

From each study, the following data were extracted: (1) authors, (2) year of publication, (3) study design, (4) purpose of the study, (5) disease condition, (6) number of patients, (7) DTI parameters used, (8) brain areas evaluated, (9) presence/absence of diffuse axonal injury (DAI), (10) cerebral functions evaluated, and (11) neurological outcomes.

## 3. Results

A scoping review was conducted, encompassing a total of 3527 studies. After the removal of 1376 duplicates, 2151 articles remained for screening. Following a rigorous eligibility assessment, 26 studies were identified as meeting the predefined inclusion criteria. Collectively, these 26 studies analyzed a total of 1169 participants, of whom 729 (62.4%) presented with moderate to severe traumatic brain injury (TBI) or diffuse axonal injury (DAI). The remaining participants served as healthy controls for comparative analysis.

### 3.1. DTI Parameters and Their Use in TBI Assessment

Among the Diffusion Tensor Imaging (DTI) parameters, fractional anisotropy (FA) was the most frequently analyzed, appearing in 25 of the 26 included studies (96.2%). Other key DTI metrics included mean diffusivity (MD), which was reported in 16 studies (61.5%), axial diffusivity (AD), in 5 studies (19.2%), radial diffusivity (RD), in 4 studies (15.4%), apparent diffusion coefficient (ADC), in 1 study (3.8%), and mean kurtosis (MK), in 1 study (3.8%). The corpus callosum was one of the most extensively examined brain regions, given its susceptibility to injury caused by angular and rotational forces. Other critical regions of interest (ROIs) included the superior longitudinal fasciculus, inferior fronto-occipital fasciculus, thalamus, basal ganglia, brainstem, and internal capsule.

### 3.2. Relationship Between DTI Metrics and Neurological Outcomes

Alterations in DTI metrics were significantly linked to neurological outcomes in individuals with TBI. Fractional anisotropy (FA) was the most frequently studied parameter, with 25 of the 26 studies evaluating its relationship with clinical, radiological, and prognostic outcomes. Seven of these studies focused on FA’s association with cognitive functions such as attention, memory, and executive functioning. Six studies assessed FA as a biomarker for axonal damage and diffuse axonal injury (DAI), while 10 studies investigated its prognostic value, demonstrating its capacity to predict mortality, disability, and recovery outcomes. Changes in other DTI parameters, including MD, AD, RD, and ADC, were also analyzed, albeit with less consistency in the findings. Increased MD was associated with edema, necrosis, and demyelination, while decreased AD was linked to axonal injury. RD increases were indicative of myelin damage. Conversely, changes in ADC were reported in only one study, and its role remains less well-defined. Notably, FA demonstrated a biphasic response: decreases in FA were linked to axonal disruption, whereas increases were interpreted as a sign of axonal recovery.

### 3.3. Brain Regions and Neurological Functions Analyzed

The role of DTI in understanding neurological outcomes was explored across multiple brain regions. The corpus callosum was the most frequently studied structure, given its critical role in inter-hemispheric communication. Damage to this area was frequently associated with poorer cognitive and motor outcomes, as highlighted in studies by Edlow et al. (2015) [2] and Graham et al. (2020) [8]. Reductions in FA within the superior longitudinal fasciculus were linked to deficits in attention and cognitive functioning. Similar trends were observed in the inferior longitudinal fasciculus and inferior fronto-occipital fasciculus, where damage was associated with impairments in the processing of emotional and visual information.

Other regions, including the thalamus, basal ganglia, and brainstem, were also examined. Reductions in FA within the thalamus were linked to deficits in learning and memory, as reported by Newcombe et al. (2015) [9]. Damage to the basal ganglia and brainstem, particularly affecting the corticospinal tract, was associated with motor coordination issues, sensory processing difficulties, and autonomic dysfunction. Injuries to these areas were further correlated with prolonged clinical recovery and the presence of post-traumatic dysautonomia.

### 3.4. Diffuse Axonal Injury (DAI) Detection Using DTI

The detection of Diffuse Axonal Injury (DAI) was a focal point in 15 of the 26 included studies. Reductions in FA were consistently identified as a hallmark of DAI, which is often undetectable using conventional MRI. DTI enabled the identification of microstructural white matter damage that is not visible on standard imaging. Changes in FA within the corpus callosum, superior longitudinal fasciculus, and brainstem were particularly indicative of DAI. In patients with grade III DAI, Munakomi et al. (2019) [10] reported a significant association between DTI findings and the duration of early clinical recovery, suggesting that DTI could be a useful tool for monitoring recovery trajectories.

### 3.5. Clinical Prognostic Relevance of DTI in TBI

The clinical and prognostic relevance of DTI in TBI was a central theme across the included studies. Reductions in FA within the corpus callosum during the early post-trauma period were predictive of poor recovery, cognitive deficits, and increased mortality risk. The timing of DTI assessments was crucial, as FA measurements conducted within the first week following injury were most effective at predicting neurological outcomes at 6-month follow-ups.

Patients with severe TBI who exhibited reduced FA in subcortical white matter were at greater risk of experiencing prolonged Post-Traumatic Amnesia (PTA), cognitive impairments, and behavioral disturbances. Studies by Andreasen et al. (2020) [11] and Cho et al. (2021) [12] highlighted that FA reductions in the uncinate fasciculus and hippocampal cingulate gyrus were negatively correlated with PTA duration and Mini-Mental State Examination (MMSE) scores. This underscores the potential utility of FA as a biomarker for categorizing patients into different prognostic groups. Additionally, DTI proved valuable in tracking recovery and neuroplasticity over time. Increases in FA observed during the months following TBI were associated with improved neurological outcomes, reflecting the processes of axonal regrowth and remyelination. This was particularly evident in studies focusing on cognitive recovery and the restoration of motor function.

For a complete overview of DTI parameter modifications and their specific role, see Table 1.

## 4. Discussion

### 4.1. Diffusion Tensor Imaging

Quantitative diffusion imaging techniques enable the characterization of tissue microstructural properties of the human brain “in vivo”, offering sensitivity to changes in microstructure due to diseases and contextually providing a suitable characterization of single-fiber distributions within a voxel. Thus, diffusion MR represents the only noninvasive technique currently used to study brain structural connectivity. DTI can image the anisotropy of white matter tracts by applying diffusion weighting in multiple different spatial directions using diffusion-sensitizing gradients. This process will ultimately yield a set of vectors that can be used to generate a structural connectivity map of the brain [2,32].

### 4.2. Current Applications of DTI in TBI

The ability to identify microstructural alterations of white matter tracts is of paramount importance in brain traumatology, as it allows us to assess the severity of the trauma and eventually predict the outcome in terms of neurological sequelae [21].

Different DTI parameters play an important role in the diagnostic field, and in future they could help clinicians to identify brain damage, leading to early treatment.

Healthy brains show diffusion limitedly to the microstructural organization of White Matter (WM) tracts, resulting in low MD/ADC values. In general, low MD/ADC is thought to be indicative of healthy/intact axons; conversely, higher MD/ADC values suggest white matter damage [33]. Dealing with FA, its reduction is directly related to axonal damage, while its increase represents a reliable index of recovery in TBI patients. The relation between FA and axonal health status makes it fundamental in the evaluation of post-TBI patients.

DTI diagnostic potentialities were first explored in rat models of TBI, focusing on structural changes in damaged brain tissue. The results obtained showed a loss of white matter integrity, namely consistent in reductions in FA and AD and increases in RD, with the latter showing a trend in reduction over the first week [32,34,35,36]. The FA reduction is an almost constant finding after both moderate and severe brain trauma and can also be related to possible cognitive deficits in acute stages [29,37]. Moreover, complex haemodynamic patterns can also be associated with DAI because of deep brain damage associated with [38,39,40,41,42]. In case of a severe trauma by shear force, a complete axotomy will be produced with the consequent fracture of microtubules/neurofilaments, water storage in the extracellular side, and blood–brain barrier (BBB) breakdown, thus leading to reduced water directional diffusion (FA reduction) [43]. Meanwhile, in the case of a simple distortion of axonal microstructures and ionic homeostasis deficit (incomplete DAI), the FA will be increased [43,44,45]. Nevertheless, it must be considered that TBI lesions are characterized by different accumulations of water (cytotoxic, vasogenic edema), and therefore fluids could confound the DTI signal [46]. To overcome this limitation, Free Water—DTI can be used to estimate Free Water Volume Fractions (FW-VF) in damaged tissues [42,43].

All these parameters are indices that help us to simply identify the location of brain damage from a radiological point of view.

The role of DTI can be expanded even in monitoring the progression of DAI. DTI may help in checking how neuroplasticity proceeds during the recovery process by showing the rearrangement of neural connections; this allows us to select cognitively compromised patients for targeted therapies, such as anti-neurodegenerative and neuroprotective treatments, overcoming conventional MRI detection possibilities [12,36]. Dealing with cerebral contusions, the peri-contusion tissue of severe head injury shows a significant vasogenic edema in terms of reduced FA and increased ADC [47]. DTI can be combined with other different techniques to improve detecting and predictive value, such as the Hybrid Diffusion Imaging (HYDI), diffusion kurtosis images (DKI), diffusion tensor tractography (DTT), and a 3D-reconstruction and configurational analysis of the bundles from the DTI data. Still, laboratory studies of serum and CSF neurofilament light (NfL) have provided useful support, along with DTI sequences, in cerebral concussion diagnosis, especially when associated with axonal damage [26,48].

### 4.3. Current Clinical Prognostic Relations of DTI in TBI

Considering the steady increase in the incidence of TBI, which is related to the increase in traffic accidents and to the aging of the population, the need to identify prognostic markers to help stratify patients with TBI, particularly moderate and severe, is crucial due to economic and social implications [49,50]. In this context, 17 of the 26 studies included used Healthy Controls (HC) to compare DTI alterations in TBI and in healthy brain tissue. The comparison confirmed the pathophysiological nature of DTI alterations [51]. The optimal timing in which to perform DTI to predict patient outcome seems to be around the first week after trauma [19]. DTI metrics can be related to mortality at 6 months and to disability rating scale (DRS), especially referring to sub-acute FA values. Moreover, both patients affected by severe TBI and aneurysmal subarachnoid hemorrhage show similar patterns [19]. Recent studies in comatose patients after TBI use both DTI and MR spectroscopy to join both structural and functional data from different techniques. It seems that a significant reduction in subcortical white matter FA with a decrease in NAA/Cr ratio in pons is related to lower rates of consciousness recovery [48]. The main WM bundle related to outcome in TBI is the corpus callosum (CC), due to its susceptibility to be injured by angular rotational acceleration. In this setting, a decrease in the FA in the CC is considered a surrogate measure of widespread Wallerian degeneration of hemispheric WM e correlates with poor prognosis. Furthermore, a correlation between FA values in the Basal Ganglia, Thalamus, and Brainstem exist with outcomes, and their evaluation can improve the detection of those patients who were incorrectly allocated unfavorable outcomes, thus influencing the decision of relatives and management [52]. When recovered, TBI patients often show an alteration of high-level cognitive functions, or behavioral changes/disorders. The assumption that TBI can disrupt brain connectivity can be proved using DTI, whose alteration in different parameters and in specific brain districts reflects the persistence or progression of pathophysiological and biomolecular mechanisms, linked to alterations in superior functions and the development of cognitive disorders. DTI alterations may be related to a late development of cognitive decline, and even to dementia [12,53]. The ability to integrate verbal/nonverbal sensory inputs from the external environment (the so-called social cognition) appears to be impaired in severe TBI, not only as a result of fronto-temporal cortex damage, but also due to the complex involvement of white matter bundles connecting inter and intra-hemispheric areas. Poor social cognition is characterized by a reduction in AF and an increase in MD in the white matter tracts [54,55]. Transient parameters such as confusion, restlessness, and post-traumatic amnesia (PTA) are widely used as criteria to classify TBI severity. A negative correlation between FA value, PTA duration, Mini Mental State Examination, and injury severity in TBI patients was found when analyzing neural structures involved in cognitive functions, with particular attention to uncinate fasciculus and the hippocampal part of the cingulate gyrus [12]. Reduced structural integrity of SLF, measured by reduced mean kurtosis (MK) and fractional anisotropy (FA) and increased mean diffusivity (MD), was associated with a reduction in overall cognitive performance [16,17]. The relation between microstructural changes revealed by DTI and the duration of PTA was investigated also by Andreasen et al. [11], whose study revealed the existence of two distinct patterns related to FA variations: one pattern was consistent with diffuse microstructural damage across the entire brain, while the other was indicative of damage to deep midline brain structures. TBI can disrupt brain communication, increasing the risk of Post-Traumatic Stress Disorder (PTSD), thus establishing a link between TBI, brain connectivity, and PTSD severity. In this regard, Mohamed et al. suggest that PTSD secondary to TBI may develop late with cognitive sequalae and brain white matter abnormalities that can be detected through DTI, and may correlate with the severity of cognitive dysfunction [53].

### 4.4. Limitations

Despite its utility, diffusion tensor imaging (DTI) has several limitations. A primary challenge is its sensitivity to head motion, which can introduce artifacts and reduce the quality of the data. Furthermore, DTI struggles to differentiate between crossing fibers within a voxel, which may lead to the misinterpretations of axonal integrity. The low Signal-to-Noise Ratio (SNR) of DTI necessitates longer scan times, limiting its feasibility in emergency clinical settings. Another significant limitation is the variability in DTI acquisition parameters across different studies, complicating the comparison of results in systematic reviews and meta-analyses. Additionally, while FA is a widely used parameter, its interpretation is not always straightforward, as changes can be attributed to various underlying factors, including edema, axonal damage, and Wallerian degeneration. Advanced imaging techniques, such as Neurite Orientation Dispersion and Density Imaging (NODDI), may address some of these challenges, but their routine use in clinical practice is still limited [5].

## 5. Conclusions

In summary, although many inherent limitations to the method still exist, DTI metrics play a significant role in the prognostication of patients with severe TBI by assessing white matter integrity, predicting functional outcomes, identifying prognostic biomarkers, and monitoring progress. Moreover, DAI after TBI is not easily detectable with conventional imaging techniques, so DTI could therefore represent the best diagnostic method for these patients. DTI provides valuable information to clinicians for individualized treatment planning, rehabilitation strategies, and counseling for patients and their families about potential outcomes following TBI [56,57,58]. The combination with complementary techniques, such as Hybrid Diffusion Imaging (HYDI), Diffusion Kurtosis Images (DKI), Diffusion Tensor Tractography (DTT), MR spectrography, or even laboratory tests could be helpful, not only for prognostic purposes, but also to increase the potentiality of this technique. Future directions demand an increasing mastery of radiological techniques to push the current boundaries of imaging, as well as further inherent clinical studies useful for developing an appropriate workflow and innovative therapeutic strategies [13,16,59,60,61,62].

## Figures and Tables

**Table 1:** Summary of papers included in the PRISMA review.

N°	Authors/Year	N° of TBI Patients	DTI Parameters	Brain Areas	DAI	Outcome	Results
1	Edlow B.L. et al., 2015 [2]	13 pts	FA, ADC, AD, RD	Corpus callosum	7 pts	Disability	FA may return to normal values in DAI. No clinical correlation is found.
2	Genova et al., 2015 [13]	42 pts + 23 HC	FA, MD, AD, and RD	Inferior longitudinal fasciculus and inferior-fronto-occipital fasciculus	no	Emotional process	DTI parameters variations in damaged WM tracts after TBI are linked to impairments in the ability to recognize facial expressions.
3	Magnoni et al., 2015 [14]	15 pts	FA	Lobar and/or cerebellar white matter, corpus callosum and brainstem	all	Global neurological functions	DTI and cerebral microdialysis can assess the extent of axonal injury.
4	Newcombe V.F. et al., 2015 [9]	12 pts	FA, MD, AD, RD	Corpus callosum, parasagittal white matter, thalamus	9 pts	Visual memory, learning task	Variation in DTI parameters are linked to variations in visual memory and learning task functions.
5	Edlow et al., 2016 [15]	11 pts + 1 HC	FA	Corpus callosum, inferior longitudinal fasciculus	yes	Global Neurological Function	Variability in acute WM FA is related to neurological outcome, and subacute FA correlated more consistently with disability rating score than acute FA.
6	Sours et al., 2016 [16]	27 pts + 27 HC	FA, MD, MK	Intraparietal sulcus	yes	Multi-sensory processing and top-down attention	Reduced structural integrity of SLF, measured by reduced MK and FA and increased MD, was associated with a reduction in overall cognitive performance.
7	O’Phelan et al., 2016 [17]	20 pts + 18 HC	FA, MD	Corpus callosum, superior longitudinal fasciculus, internal capsule, right retrolenticular internal capsule, posterior corona radiata, thalamus	N.R.	Global neurological functions	DTI quantifies the extent of damaged areas in early TBI.
8	Owens et al., 2016 [18]	20 + 20 HC	FA, MD	Medial forebrain bundle (MFB)	3 pts	Attention, working memory	Lower FA and higher MD in MFB is seen in patients with impaired attention and working memory after TBI.
9	Sener et al., 2016 [19]	43 pts + 23 aSAH	FA, MD	N.R.	4 pts	Mortality	DTI parameters, assessed at approximately day 12 after injury, correlate with mortality at 6 months in TBI and a SAH.
10	Abe et al., 2017 [20]	14 pts + 8 HC	FA, MD, AD, and RD	N.R.	7 pts	Disorders of consciousness	Modifications in FA and AD are linked to recovery from prolonged disorders of consciousness.
11	Bonanno et al., 2017 [21]	1	FA	Right hemisphere, left hemisphere	N.R.	Anosmia	Decrease in FA is related to recovery in post-TBI anosmia.
12	De Simoni et al., 2018 [22]	42 pts + 21 HC	FA, MD	Thalamus, caudate, corticostriatal tracts	N.R.	Processing speed, cognitive functions, memory, intellectual ability	DTI helps in identifying altered subcortical connectivity, linked to a large-scale network disruption and cognitive impairments after TBI.
13	Jang et al., 2018 [23]	1 pt	FA	Right corticospinal tract, left corticobulbar tract, anterior portion of both cingula, left fornical crus, right dorsolateral prefronto-thalamic tract, both lower ventral ascending reticular activating systems.	yes	Dysarthria, memory impairment, excessive daytime sleepiness	FA variations can detection DAI in patients showing several neurological deficits after TBI.
14	McDonald et al., 2018 [24]	17 pts + 17 HC	FA, MD	Corpus callosum		Gesture, facial expression, prosody, basic emotions	FA and MD alterations in CC are linked to loss of social cognition, and complex social information processing deficits after TBI.
15	Chiou et al., 2019 [25]	15 pts + 8 HC	FA	Left forceps minor and cingulum, right superior longitudinal fasciculus, forceps major, inferior fronto-occipital fasciculus, uncinate, and inferior longitudinal fasciculus	N.R.	Verbal fluency, cognitive functions	WM changes in chronic TBI (high FA) are linked to improvement in cognitive performance.
16	McDonald et al., 2019 [26]	17 + 17 HC	FA, MD	Planum temporale, corpus callosum, fornix, left temporal lobe and hippocampus, thalamus, external capsule, cerebellum, orbitofrontal cortex, frontopolar cortex, right temporal lobe	yes	Auditory localization, communication between nonverbal and verbal processes, and in memory in particular (post-traumatic amnesia), semantics and verbal recall, multimodal processing and integration, social cognition,	Loss of white matter connectivity detected by DTI alterations could predict poor social cognition after TBI.
17	Munakomi et al., 2019 [10]	17 pts	FA	Brainstem	yes	Dysautonomia	DWI values along the affected corticospinal tracts were related to the increased number of days required for early clinical recovery in patients with DAI grade III.
18	Andreasen et al., 2020 [11]	14 pts	FA, MD	Fronto-temporal, parieto-occipital, and midsagittal hemispheric white matter, as well as brainstem and basal ganglia.	yes	Post-traumatic amnesia	Two coarse spatial patterns of microstructural damage, indexed as reduction in FA, were relevant to recovery of consciousness after TBI.
19	Graham et al., 2020 [8]	55 pts + 19 HC	FA	Corpus callosum, superior corona radiata bilaterally, internal capsules, posterior corona radiata and thalamic radiation on the left	yes	Memory performance	FA variations as a measure of DAI strongly predict long-term neurodegeneration after TBI.
20	Jolly A.E. et al., 2020 [27]	117 pts + 103 HC	FA	Corpus callosum, corticospinal tract, corona radiata, inferior longitudinal fasciculi, middle cerebral peduncle	yes	Neuropsychological performance	FA quantifies entity of DAI, related to cognitive and clinical impairments after TBI.
21	Cho et al., 2021 [12]	47 pts + 47 HC	FA	In total, 48 regions of interest, among them the following: column and body of fornix, left crus of fornix, left uncinate fasciculus, right hippocampus part of cingulum, left medial lemniscus, right superior cerebellar peduncle, left superior cerebellar peduncle, and left posterior thalamic radiation	N.R.	Post-traumatic amnesia (PTA)	Negative correlation between FA value, PTA duration, Mini Mental State Examination, and injury severity in TBI patients.
22	Debarle et al., 2021 [28]	96 pts + 22 HC	FA, MD	N.R.	N.R.	Global neurological functions	Long-term normalization of MD values can predict good recovery after TBI.
23	Grassi et al., 2021 [29]	20 pts + 20 HC	MD, AD	Corpus callosum, bilateral superior longitudinal fascicles	yes	Cognitive skills	Variations in DTI parameters could predict improvement in cognitive skills after TBI.
24	Haber et al., 2021 [30]	27 pts + 14 HC	FA, MD	N.R.	yes	Global neurological functions	FA and MD can help to distinguish DAI from DVI.
25	Zaninotto et al., 2021 [31]	20 pts + 20 HC	FA, MD	Corpus callosum	yes	Depression, anxiety	DTI parameters, specifically MD and CC fibers are linked to brain injury severity in TBI, but no correlation was observed with psychiatric outcome scores.
26	Latini F. et al., 2022 [5]	6 pts + 12 athletes with repeated sport related concussion (rSRC)	AD, FA, RD	37 WM regions	N.R.	Global neurological functions and recovery	Similar regions of the left FAT, the genu of the CC, and the right ATR displayed different focal changes in both rSRC and TBI patients, reflecting possible differences in trauma and recovery mechanisms.

Abbreviations: N.R. = not reported; HC = healthy controls; pts = patients; FA = fractional anisotropy; MD = mean diffusivity; AD = axial diffusivity; RD = radial diffusivity; CC = corpus callosum; WM = white matter; DAI = diffuse axonal injury; DVI = Diffuse Vascular Injury; PTA = post-traumatic amnesia; MK = mean kurtosis; aSAH = acute subarachnoid hemorrhage; rSRC = repeated sport related concussion; WM FA = white matter regions showing decreased fractional anisotropy; MFB = the medial forebrain bundle; FAT = frontal aslant tract; ATR = anterior thalamic radiation.
